# From Street to Road: An Innovative Approach to Explore Discarded Chewing Gum as a Performance-Enhancing Modifier for Road Pavement Applications

**DOI:** 10.3390/polym13121963

**Published:** 2021-06-14

**Authors:** Nader Nciri, Namho Kim, Namjun Cho

**Affiliations:** 1 School of Industrial Design Architectural Engineering, Korea University of Technology & Education, 1600 Chungjeol-ro, Byeongcheon-myeon, Dongnam-gu, Cheonan, Chungnam 31253, Korea; nader.nciri@koreatech.ac.kr; 2 School of Energy Materials Chemical Engineering, Korea University of Technology & Education, 1600 Chungjeol-ro, Byeongcheon-myeon, Dongnam-gu, Cheonan, Chungnam 31253, Korea; njuncho@koreatech.ac.kr

**Keywords:** asphalt binder, discarded chewing gum, SARA fractions, morphology and topography, empirical tests, dynamic shear rheometer, thermal properties

## Abstract

To uncover the potential benefits of discarded chewing gum (DCG) as a performance-enhancing modifier for road pavement applications, its influence on the asphalt binder’s attributes was profoundly examined. The base AP-5 asphalt along with its specimens dosed with various fractions of DCG (e.g., 3, 6, and 9 wt%) were analyzed by Fourier transform-infrared spectroscopy (FT-IR), X-ray diffraction (XRD), thin-layer chromatography-flame ionization detection (TLC-FID), scanning electron microscopy (SEM), atomic force microscopy (AFM), thermogravimetric analysis (TGA), and differential scanning calorimetry (DSC). Brookfield viscometer, ring and ball softening point, needle penetration, and dynamic shear rheometer (DSR) tests were adopted to inspect the physical and rheological changes of asphalt cement after DCG incorporation. FT-IR disclosed that the asphalt-gum interaction was not chemical but physical in nature, whilst XRD demonstrated the existence of talc filler in DCG, which may confer the bituminous mixes with exceptional engineering properties. Iatroscan analysis evinced that the gum treatment particularly altered the aromatic and resin fractions; meanwhile, the content of saturates and asphaltenes remained relatively unchanged. SEM divulged that the DCG has a complete dissolution within the bitumen matrix, which becomes rougher due to higher dose administration. AFM revealed that the steady gum introduction amplified the size of bee-like structures, shrunk their *peri*-phase domains, and wiped out the *para*-phase domains entirely. TGA/DTGA/DSC data highlighted that the high-temperature-stable additive slightly affected the thermal properties of blends. DSR and empirical rheological tests showed that the waste gum made the bitumen less vulnerable to heat and tender, thereby boosting its resistance against fatigue cracking at intermediate service temperatures. On top of that, DCG widened the thermal window of bitumen performance grade (PG), and preserved its viscosity at standard temperatures, leading to maintaining an appropriate workability for asphalt mix. In brief, the use of discarded chewing gum as an asphalt modifier is feasible and could mitigate plastic pollution and provide durable roadways by delivering superior performance.

## 1. Introduction

The long-term performance of asphalt concrete pavements is dependent upon a number of internal and external factors, such as traffic volumes and traffic loads, material properties, subgrade soils, construction and maintenance practices, climatic and environmental conditions. The typical road pavement degrades over time and with an increasing number of load repetitions. Pavement failures occur in several common forms of distress, including but not limited to aging, raveling (i.e., aggregate loss), stripping, rutting, fatigue cracking, low-temperature thermal cracking, and reflective cracking, etc. [1].

Conventional materials utilized in the asphalt concrete mixture may perform properly relative to one distress type but fail prematurely relative to the others. For instance, asphalt concrete mixtures produced with soft asphalt binders will have poor fatigue resistance and low-temperature cracking but superior rutting potentials. On the other hand, mixtures made by using hard binders will have poor rutting resistance but superior fatigue and high-temperature cracking potential [2].

Hence, to ensure the success of building superior pavements, it is of utmost importance to modify the asphalt binder aiming at improving its performance at both high and low temperatures conditions as well as under traffic loading. Such modifications involve the addition of polymers to improve the stability of the binder and enhance its characteristics at both low and high temperatures.

In fact, it has been successfully proven that polymer-modified asphalts (PMAs) can greatly boost the road pavement’s performance. Several types of polymers have been favored, including random copolymers (e.g., ethylene-vinyl acetate (EVA) copolymers [3] and styrene-butadiene random (SBR) copolymers) [4], homopolymers (e.g., ethylene-propylene-diene (EPDM) [5], high-density polyethylene (HDPE) [6], low-density polyethylene (LDPE) [7], and polyisobutylene (PIB)) [8], and triblock copolymers (e.g., styrene-isoprene-styrene (SIS) [9], styrene-butadiene-styrene (SBS) [10], and their hydrogenated forms). PMA cannot only decrease damask and thermal sensitivity, but also increase the resistance against rutting, fatigue, and cryogenic cracks. Indeed, PMAs are frequently used in locations subjected to high stress, such as airports, race routes, truck climbing lanes, and intersections. The most common desirable features of polymeric asphalt are better cohesion and adhesion strength, higher viscosity and softening point, better elastic recovery and flexibility [11].

Governments, state transportation and environmental agencies, asphalt industries, members of academia, and contractors are striving to achieve more with less. Cost-effective, energy-efficient, and sustainable pavement solutions present a great opportunity to minimize the construction and maintenance costs of road infrastructure through the creative consumption of reused and recycled materials, as well as reduced demand on natural resources.

Waste plastic, including industrial plastics, plastic bags, and plastic bottles, poses a serious ecological threat due to its recalcitrant and non-biodegradable nature. Since 1950, more than 8.3 billion tons of plastic has been manufactured, and around 60% of that plastic has ended up in landfills or dumped directly into the natural environment, while only 9% of used plastic has been properly recycled [12,13]. Consequently, there has been a growing interest in the use of processed and recycled waste plastics in road construction. For example, it has been reported that recycled plastics can be used as modifiers or aggregate substitute in the construction of flexible pavements, thereby resulting in a reduction in their expenses [14]. The incorporation of waste plastic at small doses (ranging between 5% and 10% by weight of binder) into bituminous road construction can improve pavement stability, strength, and durability substantially [15]. Laboratory tests have shown that employing shredded waste plastic in asphalt mixtures results in higher resistance to permanent deformation (i.e., flow rutting) and greater moisture damage resistance, thereby leading to accident reduction [16].

Curiously, chewing gum is also a significant contributor to overall waste plastic, with over 100,000 tons being produced every single year [17]. Chewing gum, a tooth-friendly non-food item, is popular among teenagers, with a world market of 560,000 tons annually, which represents approximately USD 5 billion [18].

Generally, chewing gums are made up of two phases: a continuous phase known as a water-insoluble phase (WIP), and a discontinuous phase referred to as a water-soluble phase (WSP), which can be prepared by using conventional sugar (sugar chewing gums) or sugar alcohols such as polyols (sugar-free chewing gums). These phases are commonly mixed at a proportion of 3:1. Some chewing gums are often enclosed with hard or soft coating materials, which are involved in the protection of the bulking agents of gums and also in flavor release. This part constitutes the third phase.

The WIP of chewing gum is basically made of a gum-base (20–30%) that is neither inedible nor digestible; however, it is chewable while releasing sweeteners and flavors. The WIP allows the gum to be chewed for long periods of time without undergoing any compositional and structural changes. The gum-base can be manufactured either from synthetic polymers (e.g., essentially polyvinyl acetate, 15–45%) and synthetic elastomers or rubbers (10–30%) comprising a wide range of copolymers, such as isobutylene-isoprene, butadiene-styrene, polyethylene, polyisobutylene, and polyisoprene, or natural polymers such as waxes (e.g., beeswax, paraffin wax, candelilla and carnauba waxes) and plasticizers (e.g., triacetin, glycerin, propylene glycol, polyethylene glycol, lecithin, and food-grade solvents). Henceforth, this inert part of the formula makes up the support for the water-soluble components, which contain a variety of traditional ingredients, such as sweetening agents, encompassing sugar (e.g., sucrose, dextrose, and glucose syrup), sugar alcohols (e.g., polyols: xylitol, sorbitol, mannitol, maltitol, and isomalt), and artificial sweeteners (e.g., aspartame, saccharine, glycyrrhizin, and salt of acesulfame), humectants (e.g., glycerine), antioxidants, artificial colorants, flavoring agents (e.g., citrus oil, peppermint oil, and clove oil), elastomer solvents/resins (e.g., terpene resins such as polymers of α-pinene or β-pinene, glycerol, methyl, or pentaerythritol esters), emulsifiers (e.g., lecithin, glycerol monostearate, and acetylated monoglycerides), fillers (e.g., calcium carbonate, magnesium carbonate, and talc), and textural agents, etc. [19,20,21].

Even though the WSP of chewing gum is almost entirely biodegradable, the WIP is non-biodecomposable and can linger in the natural environment over long periods of time. Chewing gum can be considered as a potentially harmful pollutant to the environment and the living organisms within it. Overall, 80–90% of synthetic plastic-gum is unproperly disposed and it is the world’s second most prevalent form of street litter, after cigarette butts [22]. When uncaringly and casually tossed, it can become a sticky debris adhering to surfaces in public areas; therefore, its removal can be a costly and time-consuming exercise for local authorities. For instance, in the United Kingdom alone, local councils spend over 70 million euros as an annual cost to clean up waste gum from pavements, streets, or squares [23]. Additionally, littered chewing gum can also trap pathogens and toxins over time [24]. Although one tiny stick of gum (weighting ca. 1.5 g) may not seem like a great deal of trash, the spit-out wads add up swiftly. One environmental issues infographic speculated that 250,000 tons of the waste entering the Earth’s landfills, which are already overflowing, came mainly from the gum. To tackle this issue, some countries, such as Singapore, have already started to crack down on gum, banning it unless people have a medical reason to be chewing it [25]; meanwhile, some others have imposed fines for dropping chewing gum or recycled and processed it into versatile and potentially useful articles (e.g., pencils, travel cups, wellington boots, shoe soles, etc.) [26].

In an attempt to alleviate chewing gum mess, this research envisions using discarded chewing gum (DCG) as a modifier agent to enhance the engineering properties of road pavements.

## 2. Materials and Methods

### 2.1. Preparation of Discarded Chewing Gum (DCG)-Asphalt Blends

The base asphalt AP-5 (PG 70–22), graciously provided by the Korea Federation of Ascon Industry Cooperative R&D Center (Osan-si, Korea), was used in this study. The physicochemical properties are condensed in Table 1.

**Figure 1:**
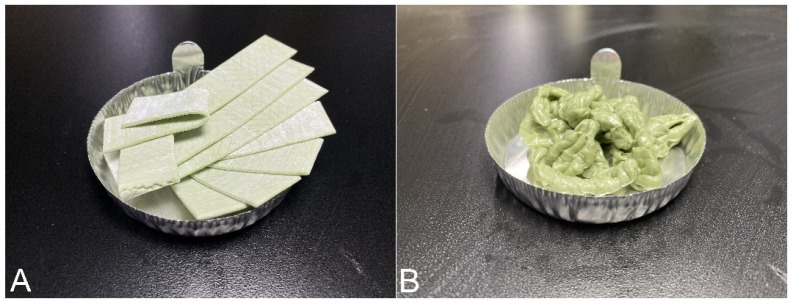
Commercial chewing gum (**A**) and its fresh discarded (**B**) specimens.

One commercially popular available chewing gum (ingredients: white sugar, gum base, artificial sweeteners (e.g., acesulfame potassium and sucralose), crystalline glucose, corn syrup, artificial flavors (e.g., peppermint flavor), emulsifier, glycerin, D-sorbitol solution, grape concentrate, pineapple concentrate, mixed formulation (e.g., green tea powder, emulsifier, and acesulfame potassium), green dye coloring, gardenia blue, safflower yellow, and chlorophyll/green tea powder 0.2%) in the Korean market was used in this investigation. The fresh discarded chewing gum (DCG) samples, shown in Figure 1, were donated directly by adult human volunteers in Korea University of Technology and Education and stored hermetically in sealed containers at 4 °C. These samples were hygienically collected from various individuals in the Department of Architectural Engineering after they had chewed the gum for 30 min at least 1 h after eating or drinking. This step was done to ensure the obtention of pure gum free to the greatest extent possible of any food residues and gum water-soluble substances.

Before being incorporated into the base asphalt, the DCG specimens were dried in a connective vacuum oven at 100 °C for 24 h. Characteristics of DCG are given in Table 2.

The production of DCG-modified asphalts was carried out in the Construction Material Laboratory using a L5M-A mixer (Silverson Machines Inc., East Longmeadow, MA, USA), with a high-shear speed of 3000 rpm at a temperature of 180 °C. Aluminum cans of 1000 mL volume were filled with 600 g of the base asphalt (AP-5 asphalt) and preheated to fully fluid state. Upon reaching 175 °C, the discarded chewing gum was added stepwise into the binder at concentrations of 3, 6, and 9 wt% by the weight of the binder. Careful consideration should be given during stirring, as instantaneous foam formation can take place. After the temperature was raised to 180 °C, the blend was continuously stirred at this temperature for 2 h to ensure a proper homogenous mixing [10,27,28]. Subsequently, the DCG-bitumen combination was removed from the can, gently divided into small metal containers, and sealed immediately with aluminum foil. Finally, the specimens were left to cool overnight at room temperature (ca. 25 °C) and stored for further tests. The technique adopted in the production of blends allowed the binder to be subjected to relatively high temperatures and air for a certain period of time, which caused its hardening. To accurately evaluate the discarded chewing gum’s influence, the base asphalt AP-5 was also subjected to the same treatment as the DCG-asphalt mixtures.

### 2.2. Fourier-Transform Infrared Spectroscopy (FT-IR)

The FT-IR was recorded on a Hyperion 3000 FT-IR Spectrometer (Bruker Optics, Ettlinger, Germany) with a spectral resolution of 1 cm^−1^, a wavenumber range between 4000 and 650 cm^−1^, and an average of 30 scans per sample. The bituminous samples as well as DCG were examined using a thin disk of the sample mixed with KBr (i.e., potassium bromide). FT-IR was conducted to reveal the changes in the molecular components and structures of the base asphalt (AP-5 asphalt) after treatment with different doses of discarded chewing gum (e.g., 3, 6, and 9 wt% DCG).

### 2.3. X-ray Diffraction Spectroscopy (XRD)

To further illuminate the effect of discarded chewing gum (DCG) with its possible existing fillers on the microcrystalline phase of base AP-5 asphalt, X-ray diffraction was carried out using a Bruker AXS D8 Advance Diffractometer (Bruker AXS GmbH D8 Advance, Karlsruhe, Germany) with CuKα radiation (λ = 1.54005 Å, 40 kV and 40 mA) at ambient temperature (ca. 25 °C). The diffraction (2θ) was monitored from 10° to 90° at a 1°/min scan rate with a 0.05 step size.

### 2.4. Thin-Layer Chromatography-Flame Ionization Detection (TLC-FID)

The unmodified and DCG-modified asphalt samples as well as the discarded chewing gum (DCG) were analyzed by TLC-FID analyzer Iatroscan MK6s (Iatroscan analyzer, Iatron Laboratories Inc., Tokyo, Japan). A TH-10 Mk IV instrument equipped with a flame ionization detector and interfaced with an electronic integrator (Perkin-Elmer LCI-100) was used for rod scanning and quantification. Silica rods, Chromarods-SIII (pore diameter 60 Å, particle size 5 μm), were used for TLC. Before sample application, the chromarods were passed several times through the FID to remove contaminants and impurities, and to obtain a constant activity of the silica layer. Pure grade hydrogen (160 mL/min) and air velocity (2 L/min) supplied by a pump were used for the detector. A 2% (*w/v*) sample solution (asphalt/dichloromethane) was prepared and 1 μL of the sample solution was dropped at silica gel chromarod with a 5 μL Drummond microdispenser (Drummond Scientific, Broomall, PA, USA). Then, the chromarod was successfully soaked and extended in three different tanks containing *n*-hexane (100%, to elute saturates), toluene (100%, to elute aromatics), and dichloromethane/methanol (95/5 by volume, to elute resins) solvents, respectively. Each component in the chromarod generates organic ions when burned by a hydrogen flame and are converted to a different current intensity, which could be quantitatively detected by FID. For each quartz rod, the four peak areas were integrated at the lowest point before and after each peak, after which the percent concentrations for saturates, aromatics, resins, and asphaltenes (SARA) were calculated. The frame with 10 rods was placed in the drying chamber at 85 °C for around 2 min to evaporate the residual solvent. The scanning speed for FID was 30 s/scan and five parallel specimens were applied for each asphalt layer. There were 5 parallel specimens for each sample. The TLC-FID analyses were performed in quintuplicate.

### 2.5. Scanning Electron Microscopy (SEM)

The JSM-6010LA scanning electron microscope (SEM) (JSM-6010LA, JEOL Ltd., Tokyo, Japan) produced by JEOL Ltd. of Japan was employed to investigate the impact of different concentrations of discarded chewing gum (e.g., 3, 6, and 9 wt% DCG) on the microstructure and surface morphology of the base asphalt (AP-5 asphalt). The asphalt specimens were cryogenically fractured in liquid nitrogen to insure a sharp brittle fracture. The exposed surface (cross-section) of each sample was sputter-coated with a thin gold film (i.e., 12 nm) to avoid electrostatic charge build-up during diagnosis. The acceleration voltage of the electron beam was 5 kV. The probe current (spot size) was varied between 4 and 6 nA, and the working distance (WD) was around 10 mm. The SEM micrographs were obtained under the magnification ×3000.

### 2.6. Atomic Force Microscopy (AFM)

To investigate the influence of discarded chewing gum (DCG) on the surface microstructure and micromechanical behavior (e.g., friction) of base AP-5 asphalt, atomic force microscopy (AFM, XE-100, Park Systems, Suwon, Korea) force mapping was performed with a Bruker Dimension Icon atomic force microscope in PeakForce mode. The different asphalt samples were heated to a flow state at 140 °C, carefully dropped onto a 25 mm × 75 mm glass microscope slide, and stored in a Petri dish to avoid external contamination. The samples were gently annealed at room temperature (ca. 25 °C) and ambient air (relative humidity (RH) 50%) for a minimum 24 h before scanning. To collect AFM images, a Bruker Dimension 3000 was employed with a soft-crystalline silicon probing tip (Type: PPP-CONTSCR, NanoSensors™, Neuchâtel, Switzerland) in tapping mode. The tip possesses a diameter in the order of 10 nm (at the end of a cantilever), a laser diode set to reflect off of the back of the cantilever, and a position-sensitive photodetector. Before proceeding with scanning, the sensitivity of the photodiode was calibrated by registering a force–distance curve with a cantilever on a smooth and non-compliant nanocrystalline diamond thin film in air, in order to avoid sample deformation effects. Afterwards, the thermal vibration noise was analyzed to determine the normal and lateral stiffness of the cantilever (C_n_ = 0.02 N/m and C_1_ = 3.97 N/m) [29]. The topography and lateral force images were simultaneously collected. AFM images were acquired at several locations on the sample surface with a normal force-value F_n_ = 0 nN, to get a clear picture of the bitumen specimen. The friction force maps were measured according to the following formula: F_f_ = (F_l,fwd_ − F_l,bwd_)/2, where F_l,fwd_ and F_l,bwd_ are the lateral force signals recorded in the forward and backward direction, respectively. The unit of the lateral force signals was obtained by converting the lateral voltage of the photodiode into a unit of force following this formula: F_l_ = S × C_l_ × V_l_ × (3 h/2 L), where h is the tip height, L is the length of the cantilever, S is the sensitivity of the photodiode, and V_l_ is the lateral voltage of the photodiode [29].

### 2.7. Thermogravimetric Analysis (TGA/DTGA)

To study the effect of different fractions of discarded chewing gum (e.g., 3, 6, 9 wt% DCG) on the thermal properties of the neat asphalt (i.e., AP-5 asphalt), TGA analyses were carried out using a thermogravimetric analyzer (TGA Q500, TA Instruments, New Castle, DE, USA). Measurements were conducted by heating ca. 10 mg of bitumen sample from 30 to 1000 °C, at a heating rate of 20 °C min^−1^ and under nitrogen atmosphere (150 mL/min N_2_ flow). The TGA test was repeated three times to ensure reproducibility and repeatability.

### 2.8. Differential Scanning Calorimetry (DSC)

The DSC technique, performed using a PerkinElmer 8000 DSC (PerkinElmer, Waltham, MA, USA), was utilized to record and probe the thermal effects of various proportions of discarded chewing gum (e.g., 3, 6, 9 wt% DCG) included in the base asphalt (AP-5 asphalt) through the cooling–heating cycles. A small amount of asphalt sample (ca. 15–20 mg) is placed in an aluminum crucible and sealed under an inert nitrogen atmosphere to minimize oxidative degradation. Another crucible without a sample was used as reference. To guarantee a steady initial reading, the sample was heated from room temperature ca. +25 to +50 °C at a scanning rate of 10 °C/min and kept for 10 min. Subsequently, the sample was cooled down at a heating rate of 20 °C/min to −90 °C, followed by heating to +150 °C at the same rate. To remove all fingerprints of the thermal history from asphaltaneous samples, the cooling and heating processes were executed. After accomplishing the first scan, the sample was subjected to a swift quenching from +150 to −90 °C and remained at that temperature for about 10 min, followed by reheating at a heating rate of 20 °C/min to +150 °C. The different calorimetric parameters such as phase transition temperatures (T_g_, T_m_) and enthalpy changes (∆H) were collected directly from the second heating scan. All DSC tests were repeated three times for reproducibility.

### 2.9. Conventional Binder Tests (Penetration, Softening Point, and Viscosity)

The base asphalt (AP-5 asphalt) and asphalt samples containing various fractions of discarded chewing gum (e.g., 3, 6, 9 wt% DCG) were subjected to the following conventional tests according to ASTM standards: the penetration test was conducted using Humboldt Mfg Electric Penetrometer (Humboldt Mfg. Co., Elgin, IL, USA) in accordance with the procedure laid down in ASTM D5 [30]. This test determines the hardness-consistency of bitumen by measuring the depth in tenths of a millimeter (dmm), to which a standard needle is allowed to vertically sink into a binder sample under 100 g load for 5 s at room temperature (normally 25 °C). The softening point test was performed with Ring & Ball-Apparatus RKA 5 (Anton Paar GmbH, Ashland, VA, USA) according to ASTM D36 [31]. Softening points were employed to determine the temperature at which a phase change occurs in the binder. This temperature is known as the ring and ball softening temperature (T_R&B_). The standard rotational viscometer test was conducted according to standard procedure detailed in ASTM D4402 [32] using Brookfield DV III Rheometer (Brookfield, Middleboro, MA, USA) equipped with a coaxial cylindrical spindle (shear rate 6.8 s^−1^) and a thermal chamber (Thermosel ^TM^, Brookfield, Middleboro, MA, USA). The viscosity was determined by sensing the torque required for rotating the SC4-27 spindle at a constant rotational speed 20 rpm upon immersing it in 10 mL of fluid asphalt sample at 135 °C.

### 2.10. Temperature Susceptibility (TS)

To elucidate the impact of various doses of discarded chewing gum (e.g., 3, 6, 9 wt% DCG) on the temperature susceptibility of the base asphalt (AP-5 asphalt), the penetration index (PI) and penetration viscosity number (PVN) were investigated. Higher PI and PVN values are indicators of a binder that is less vulnerable to temperature changes (i.e., low temperature-susceptibility) and vice versa (i.e., indicating the asphalt has higher temperature susceptibility) [33]. A low-temperature-susceptible bitumen will acquire a reasonably high viscosity at high service temperatures and a reasonable flexibility at low service temperatures. It should be stressed that these approaches are only rough indicators for the rheological performance of unmodified and DCG-modified binders in an actual hot-mix pavement. To gain a more accurate correlation with road pavement performance, dynamic shear rheometer (DSR) data for the investigated binders were required.

The PI is calculated from Equation (1):

(1) $$\mathrm{PI}=\frac{1952-500\log P-20\times \;\mathrm{SP}\;}{50\log P\;-\;\mathrm{SP}\;-120}$$

where P: penetration at 25 °C, 100 g, 5 s (dmm); and SP: softening point (°C).

The PVN is determined from Equation (2):

(2) $$\mathrm{PVN}=-1.5\;\frac{4.2580\;–\;0.7967\;\log\;P\;-\;\log\;V}{0.7591-0.1858\;\log\;P}$$

where P: penetration at 25 °C, 100 g, 5 s (dmm); and V: kinematic viscosity at 135 °C (cSt).

### 2.11. Laboratory Asphalt-Aging Procedure

In this research project, the virgin asphalt (AP-5 DCG 0 wt%) and its specimens loaded with various fractions of DCG (e.g., 3, 6, and 9 wt%) were subjected to artificial aging such as by means of a rolling thin-film oven (RTFO) and pressure aging vessel (PAV). According to ASTM D8272-19 [34], the different asphaltaneous samples (each weighting ca. 35 ± 0.5 g) were short-term aged using RTFO (Model CS325, James Cox & Sons, Inc., Colfax, CA, USA) at a constant temperature of 163 ± 0.5 °C for 85 min in a rolling oven under a constant airflow (4000 mL/min). This procedure was designed to simulate manufacturing and placement aging. Based on ASTM D6521-13 [35], the diverse RTFO-aged bitumen samples (each weighting ca. 50 ± 0.5 g) underwent long-term aging using PAV (PAV3, Applied Test Systems LLC, Butler, PA, USA) at a constant temperature of 100 °C for 20 h under 2.1 ± 0.1 MPa of air pressure. This procedure was executed to simulate in-service oxidative aging over a 7- to 10-year period. After treatment, the samples were removed and degassed in a VDO 81-PV2610 (i.e., vacuum degassing oven, Nova Measurements LLC, Atlixco, Puebla, México) at 170 °C for 30 min to remove all air bubbles created during accelerated oxidative aging of asphalt cement by the PAV3. Finally, all samples were stored for further testing in hermetically sealed tin containers at an ambient temperature (ca. 25 °C).

### 2.12. Dynamic Shear Rheometer (DSR) Test

A dynamic shear rheometer (DSR) from ThermoFisher (Thermo Scientific^TM^ HAAKE^TM^ MARS^TM^ Rheometer, ThermoFisher Scientific, Newington, NH, USA) was used to examine the direct effect of discarded chewing gum (DCG) on the viscous and elastic behaviors of the base asphalt (AP-5 asphalt) at high temperatures (e.g., 46~82 °C) and intermediate temperatures (e.g., 4~40 °C) to control permanent deformation (i.e., rutting) at high temperatures and fatigue cracking at intermediate temperatures, respectively. This diagnosis was performed according to ASTM D7175 [36]. The rheological characterization of unmodified and DCG-modified asphalt samples was conducted at a loading frequency of 10 rad s^−1^ and expressed in terms of complex shear modulus (i.e., stiffness), G*, and the phase angle, δ (i.e., elasticity). The SHRP asphalt binder specification defines G*/sin δ as permanent deformation (i.e., rutting) and G*.sin δ as fatigue cracking. The geometrics with gaps at test temperatures were executed with 8-mm diameter parallel plates with a 2-mm gap below 45 °C and 25 mm diameter parallel plates with a 1 mm gap above 45 °C.

## 3. Results and Discussion

### 3.1. Fourier-Transform Infrared Spectroscopy (FT-IR)

Fourier-transform infrared spectroscopy (FT-IR) was employed to probe the impact of various concentrations of discarded chewing gum (e.g., 3, 6, 9 wt% DCG) on the molecular structure and chemical composition of AP-5 asphalt.

**Figure 2:**
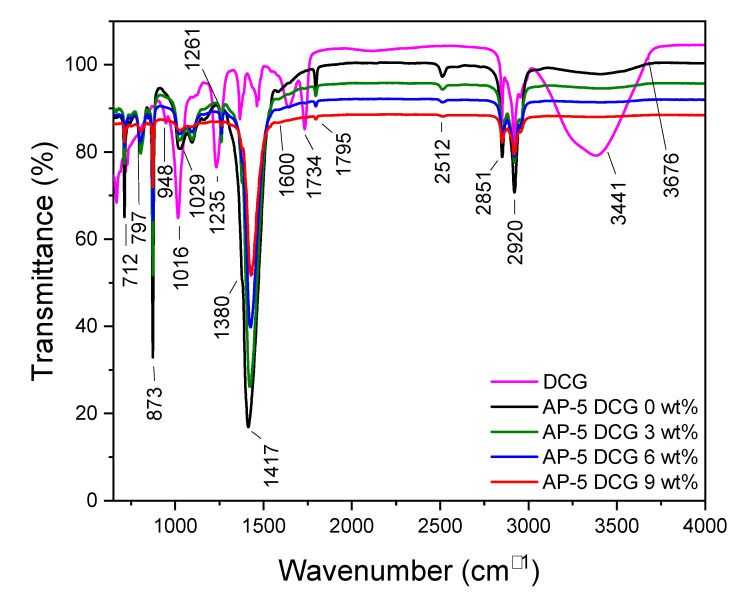
FT-IR spectra of fresh DCG, base AP-5 asphalt, and its specimens loaded with various fractions of DCG (e.g., 3, 6, and 9 wt%).

Figure 2 shows the FT-IR spectra of base AP-5 asphalt, fresh DCG, and DCG-modified asphalt specimens. The IR spectrum of straight binder (i.e., AP-5 DCG 0 wt%) shows the N–H/O–H stretching and bending bonds which are detected within the 3100–3500 cm^−1^ region as a broad-shaped and medium-intensity signal. This peak becomes wider and larger with the DCG specimen. The strong peaks that appeared around 2851 and 2920 cm^−1^ are ascribed to the C–H symmetric stretching (–CH_2_) and the C–H asymmetric stretching vibrations (–CH_3_ and –CH_2_) of hydrocarbon chain segments, respectively.

The numerous spectral bands identified at 2512, 1795, 873, and 712 cm^−1^ are undoubtedly caused by an undesirable external contamination (happening during experiments) with limestone mineral filler [37].

No remarkable absorption peak of the carbonyl group (C=O) was observed at 1700 cm^−1^, which might suggest a lower degree of oxidation. The tiny shoulder at 1600 cm^−1^ is caused by the conjugate double bonds C=C in benzene rings (aromatic hydrocarbons) and C–H bonds stretching vibrations. The two peaks at 1417 cm^−1^ and 1380 cm^−1^ are assigned to the C–H asymmetric bending vibrations of –(CH_2_)_n_– and –CH_3_, respectively. These peaks are common to both the waste gum (made up more probably with polyisobutylene (C_4_H_8_)_n_ as an elastomer and polyvinyl acetate (C_4_H_6_O_2_)_n_ as a binder) and the bitumen. The peaks at 1029 and 725.14 cm^−1^ are due to the S=O bond and the rocking vibration (i.e., bending with torsion) characteristic of the CH_2_ band, respectively. The sharp peak in frequency at 1261 cm^−1^ corresponds to the presence of C–C (=O)–O– stretching vibration for ester group.

The FT-IR spectrum of discarded chewing gum (DCG) is depicted in Figure 2. The least prominent band occurred around 1734 cm^−1^ and is attributed to the ester carbonyl functional group –C=O stretching vibrations of acetate groups in polyvinyl acetate (PVAc). The second least strong band appeared at 1235 cm^−1^ corresponds to the asymmetric stretching mode of –C–(C=O)–C– ester group, followed by two distinct lower wavelengths with the maximum at 1016 cm^−1^ and the less intense one at 948 cm^−1^ [38]. Additionally, the other characteristic absorption bands of PVAc can be observed at frequencies of 3280 cm^−1^, 2916 cm^−1^, 1462 cm^−1^, and 1366 cm^−1^, with a C–H rocking vibration at 797 cm^−1^, 632 cm^−1^, and 604 cm^−1^ (the two last wavelengths are not shown in the spectrum) [38]. Similar peaks are effectively noticed in the asphalt-polymer blends.

The infrared spectrum of DCG curiously shows the characteristic bands of talc powder (Mg_3_Si_4_O_10_(OH)_2_) at 1014 cm^−1^ which are assigned to Si–O stretching vibration, and at 3441 cm^−1^ and 3676 cm^−1^ which are allocated to the stretching vibrations of hydroxyl group O–H linked to Si (Si–OH) and Mg (Mg–OH), respectively [39].

No apparent new absorption band was formed after the inclusion of the polymer into the asphalt binder, thereby indicating that there no obvious chemical reaction took place.

### 3.2. X-ray Diffraction Spectroscopy (XRD)

The X-ray diffraction spectroscopy (XRD) was conducted to elucidate the influence of diverse amounts of discarded chewing gum (e.g., 3, 6, 9 wt% DCG) on the crystallographic structure of base AP-5 asphalt as well as to possibly identify the different fillers (e.g., calcium carbonate, magnesium silicate (talc)) employed in the chewing gum manufacturing; keeping in mind that these fine-grained mineral particles hold a great potential for empowering the performance of asphalt mixtures [40,41,42,43].

**Figure 3:**
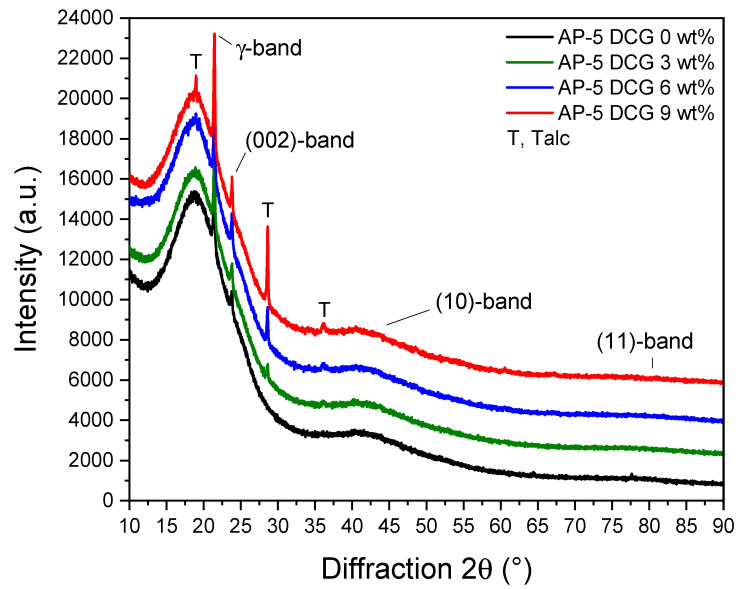
XRD patterns of base AP-5 asphalt and its samples including different doses of DCG (e.g., 3, 6, 9 wt%).

Figure 3 depicts the XRD patterns of unmodified and DCG-modified asphalt samples. It illustrates that the plain bitumen (i.e., AP-5 DCG 0 wt%) is completely amorphous. The XRD pattern of neat asphalt shows a very noticeable band at 2θ = 21°, commonly known as gamma band (γ), which arises from the packing distance of aliphatic chains or the layers of condensed saturated rings. Another remarkable band called graphene or (002)-band, generally superimposed with the (γ)-band, occurred at around 2θ = 23°. This asymmetric peak originates from the diffraction of X-rays of stacks of aromatic molecules. The two weak (10) and (11) bands, or (100) and (110) reflections come from the in-plane structure of aromatics [44]. These peaks appear at 2θ = 40° and 2θ = 80°, respectively. The (10) peak designates the degree of condensation of the aromatic rings (i.e., size of the aromatic layer) [44].

A close inspection of the bunch XRD charts confirms the presence of soapstone or talcum clay minerals (Mg_3_Si_4_O_10_(OH)_2_) as either a texturizing/anti-sticking agent or filler residue in the gum base and asphaltaneous compounds, respectively; finding which is supported by the work of Zhao and co-researchers [45]. For safety purposes, the DCG specimen was not subjected to XRD analysis. The XRD spectra of binder-gum blends display the several additional talc peaks at approximately 2θ = 19°, 28°, and 36° and two already-existing amorphous humps; a major one centered around 20° and a broad minor one located at 40°, thereby revealing that the mixtures still preserve their amorphous microstructures and the DCG did not affect them.

Referring to Figure 3, when the waste polymer content in bitumen reaches 3 wt% or 9 wt% in weight, the peak intensity drops marginally. This decline in crystallinity magnitude is mainly contributed to the random dispersion of polymer in the binder medium. However, as the waste of polymer content increases to 6 wt%, the peak intensity rises and exceeds slightly the value of the pure bitumen. As stated in the literature [44,46,47], X-ray diffraction analysis is a powerful nondestructive technique that can be used to characterize the physicochemical properties of bitumen using peak intensity and peak area, etc. The peak intensity reflects the relative strength of diffraction. In other terms, the lower peak intensity mirrors the less strength of diffraction and better quality of polymer content, since the polymer network possesses an interlaced form in the binder matrix. Hence, the proportion 9 wt% of gum has a better quality than the other doses, while the 6 wt% DCG is less favorable than other proportions.

### 3.3. Thin-Layer Chromatography-Flame Ionization Detection (TLC-FID)

**Figure 4:**
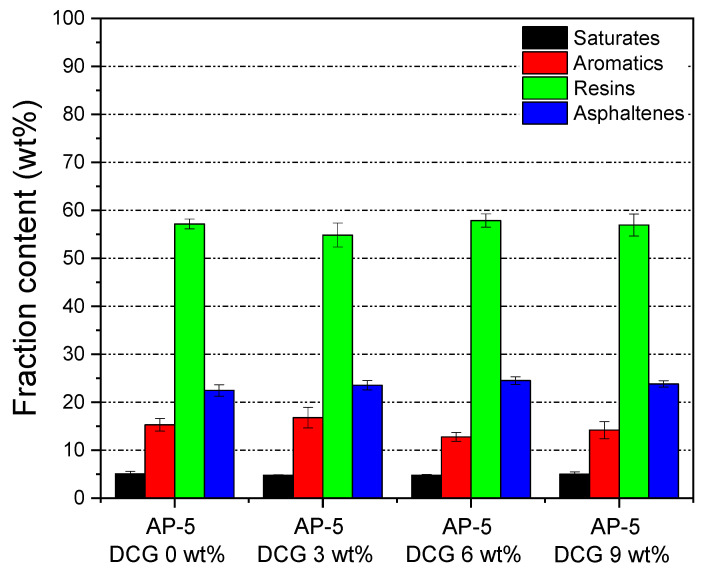
Impact of various dosages of DCG (e.g., 3, 6, 9 wt%) on the SARA generic fractions of base AP-5 asphalt.

Thin-layer chromatography-flame ionization detection or TLC-FID allows the complete screening of chemical classes as well as their quantification. To gain an insight into the effect of various dosages (e.g., 3, 6, 9 wt% DCG) of discarded chewing gum on the fractional composition SARA (i.e., saturates, aromatics, resins, and asphaltenes) of base AP-5 asphalt, TLC-FID was conducted accordingly. The test results are presented in Figure 4. For the unmodified asphalt (i.e., AP-5 DCG 0 wt%), it can be readily noticed that the most prominent groups are resins (57.16 wt%), followed by asphaltenes (22.48 wt%), aromatics (15.30 wt%), and saturates (5.08 wt%). One the other side, DCG-modified asphalt samples (i.e., AP-5 DCG 3, 6, and 9 wt%) exhibit significant differences in the group composition due to the presence of the modifier. The average contents of aliphatic (e.g., polyisobutylene (PIB) as elastomer), aromatic (e.g., artificial colorants such as safflower yellow, green tea polyphenols, etc.), and naphthenic hydrocarbons/resins (e.g., glycerin as plasticizer, polyvinyl acetate (PVAc) as binder, fats, oils, etc.) compounds in discarded chewing gum (DCG) specimen are 17.98, 22.93, and 59.08 wt%, respectively. As can be observed from Figure 4, the addition of DCG to the virgin bitumen affected its aromatics and resins more than the other fractions, whereas the content of saturates and asphaltenes did not show any significant changes. At various doses, it is obvious that the gum waste reacted differently with the asphalt constituents. For instance, 3 wt% DCG, which represents the opposite effect of 6 wt% DCG, reduced the resin concentration and increased the aromatic amount. On the other side, the pure asphalt modified with 9 wt% DCG noted only a slight decrease in its aromatics, associated with sluggish growth in the proportion of asphaltenes. Neither increasing/decreasing nor a stable trend can be established at this stage and further multiple higher shots of waste gum along with a deep statistical analysis might be needed to clearly see the direction of behavior change in the SARA composition of base AP-5 asphalt. The complexity of discarded chewing gum as a multicomponent material comprising several elements with diverse chemical, physico-rheological, and thermal properties could stand partially behind the poor correlation of data. The current divergence found between the chemical responses of asphalt specimens toward the additive treatment could also be translated later by a divergence in thermal and physico-rheological responses as well.

### 3.4. Scanning Electron Microscopy (SEM)

To develop further understanding regarding the microstructural and micromorphological changes of AP-5 asphalt in response to the discarded chewing gum (DCG) treatment, scanning electron microscopy (SEM) with energy-dispersive X-ray spectroscopy (EDXS) was carried out.

**Figure 5:**
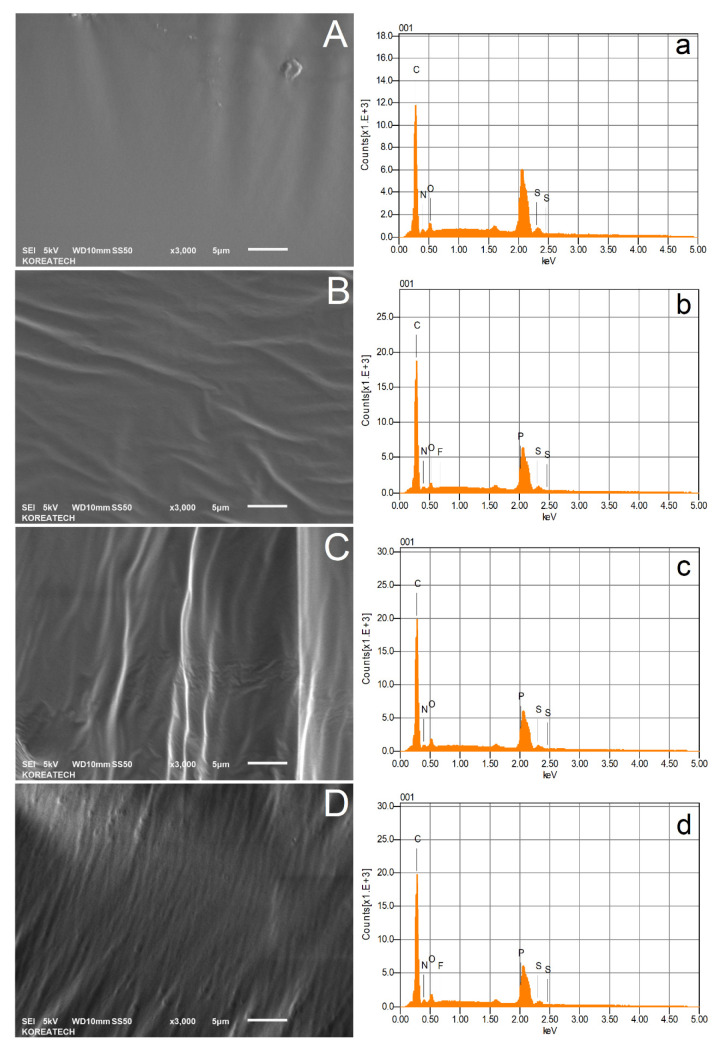
Scanning electron microscope (SEM) micrographs with their corresponding EDXS spectra of unmodified and DCG-modified asphalts taken at ×3000 magnification. (**A**,**a**) AP-5 DCG 0 wt%; (**B**,**b**) AP-5 DCG 3 wt%; (**C**,**c**) AP-5 DCG 6 wt%; (**D**,**d**) AP-5 DCG 9 wt%.

According to Figure 5A, it can be seen that the virgin AP-5 asphalt (i.e., AP-5 DCG 0 wt%) exhibits a very uniform, smooth, and homogenous surface structure. SEM micrographs of asphaltaneous samples compounded with various portions of discarded chewing gum (e.g., 3, 6, and 9 wt% DCG) are given in Figure 5B–D, respectively. When the dose of DCG increases, the polymer fraction still always unnoticeable. The thermally stable DCG particles are apparently well dispersed in the asphalt matrix. To add more credibility to this claim, additional compatibility tests (e.g., fluorescence microcopy) are strongly recommended.

Simultaneously, quantitative EDXS microanalysis, shown in Figure 5a–d, revealed an increase in carbon and oxygen proportions, a slight decline in sulfur concentration, and a stable nitrogen value. It can be curiously seen that the mixes comprise the phosphorus (P) element which may come from the lecithin used as an emulsifier in the gum processing. Likewise, a prominent peak in the 2.1 keV region announced the presence of gold (Au), which was utilized as a thin sputter-coating layer on the sample before proceeding with SEM.

At a low gum concentration of 3 wt% DCG, no sign of any rubbery particles was seen in the continuous asphalt phase under a magnification of ×3000 (Figure 5B). In this case, the fairly uneven and wrinkled asphalt cement constitutes the continuous phase of the system, where the discontinuous phase represented by the polymeric fraction is disseminated through it. The dark phase in SEM photograph may indicate the compatible polymer–asphalt mixture and reflects the instant SARA (i.e., saturates, aromatics, resins, and asphaltenes) results obtained by means of TLC-FID technique.

The genesis of microstructures starts to take place eventually when the concentration of DCG reaches 6 wt% (Figure 5C). At this dose, the rubber phase abruptly disturbed the matrix of the whole system, and the DCG/asphalt blend displays a rippled topography.

It can be admitted that a great dispersion of polymer is accomplished by using 3 wt% DCG. The minimum polymer concentration required to attain an equilibrium state depends, to an extreme degree, on the binder rather than on DCG. In this case, the gum amount is considerably low for the polymer phase to dominate the matrix system.

Upon reaching 9 wt%, the DCG most likely governs the whole system properties. A high level of non-compatibility can be readily perceived through the wide polymeric agglomerations scattered randomly throughout the rough bitumen body (Figure 5D). This incompatibility can effectively damage the engineering properties of AP-5 asphalt such as tenacity and toughness. Due to the variation in the properties such as structure, polarity, and molecular weight, there is a chemical dissimilarity between bitumen and discarded chewing gum. The micromorphology and microstructure are the consequence of the mutual interaction between the rubber and binder, and hence are mainly affected by the characteristics of the polymer (e.g., concentration, chemical structure, and molecular weight distribution) and the characteristics of the base binder (e.g., asphalt source and grade, chemical constituents, structure (colloidal state), and rheology (viscoelasticity)).

### 3.5. Atomic Force Microscopy (AFM)

To shed light on the impact of discarded chewing gum (DCG) on the microsurface structure and micromechanical properties of base AP-5 asphalt, contact mode AFM measurements were conducted. It should be noted that doses over 3 wt% of additive generated unsatisfied data due to the extreme stickiness of the cantilever tip to the gummy sample. Therefore, minor shots were adopted accordingly.

**Figure 6:**
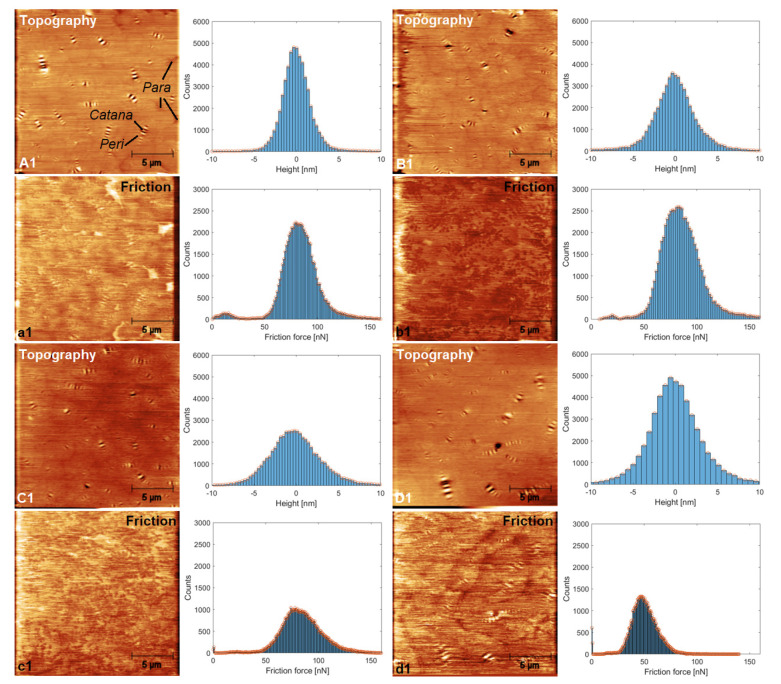
AFM topography and friction images with their corresponding histograms on the right side highlighting the influence of DCG on base AP-5 asphalt micromorphology and friction. (**A1**,**a1**) AP-5 DCG 0 wt%; (**B1**,**b1**) AP-5 DCG 1 wt%; (**C1**,**c1**) AP-5 DCG 2 wt%; (**D1**,**d1**) AP-5 DCG 3 wt%.

The AFM topographic pictures of the plain bitumen (i.e., AP-5 DCG 0 wt%) along with its specimens blended with various fractions of DCG (i.e., 1, 2 and 3 wt%) are given in Figure 6A1, B1, C1, D1, respectively. The corresponding friction pictures are illustrated in Figure 6a1, b2, c2, d2, respectively. It can be seen clearly from Figure 6A1 that the virgin bitumen reveals the presence of three distinct microstructural zones. A wrinkled “*catana*” zone with valleys and hills known commonly as a “bee” structure can be observed. In Greek, “*cato*” means low, while “*nano*” signifies high. The “*catana*” zone is encircled by a dense “*peri*” phase. In Greek, *peri* refers to around. The last zone is called “*para*”, which means neighboring. This phase is relatively softer compared to the other domains and could occasionally include some tiny quasi-spherical shaped-dots recognized as “*sal*” phase or salt in Latin [48].

The introduction of 1 or 2 wt% DCG into the straight binder gave birth to some baby bees and induced eventually a reduction in the size and amplitude of parent bees with a considerable shrinking of *peri*-domains (Figure 6B1,C1). Upon the incorporation of 3 wt% of waste gum, the bees demonstrated a drastic amplification in size as well as in length (Figure 6D1). The boundaries separated the several cells enclosing the bees are barely distinguishable, pointing out that the *para*-phase is almost drained out (i.e., solvent region). The slight upsurge in wax and asphaltenes level and their mutual interaction with aromatics and resins could be responsible for the aforementioned changes [49,50].

Surface roughness data are portrayed in Figure 6 (Counts = f (Height [nm])). Usually, as the bee-like structures become larger and/or more numerous, roughness value has a great tendency to increase. Additionally, with more gum waste administration, the bitumen evidently developed a rough and sticky surface texture especially with 3 wt% dose, a finding which is fostered by the SEM data.

Figure 6 equally presents the friction force data as a compilation of images and histograms split between the untreated binder and binder treated with various dosages of DCG (e.g., 1, 2, and 3 wt%). These data were received by employing a lateral force mode of AFM. The friction or dragging force, which symbolizes the resistance to flow, is stronger when crossing the bright and clear areas and becomes weaker in the dark areas. The bright domains typically belong to the *para*-phase structure which governs the overall physico-mechanical properties of asphalt mixtures. Interestingly, it can be realized that the multiple histograms gave rise to two non-Gaussian bands, one minor located below 25 nN, probably assigned to *para*-phase domains, and one major above 25 nN, presumably accounting for *peri*-phase domains. The non-Gaussianity directly probes the existence a multi-phased compound with diverse micro-textures. Noticeably, the incremental addition of the modifier discernibly reduced the intensity of these peaks and moved them downward, thereby demonstrating that the dragging force was substantially diminished as a result of the excessive adhesiveness imposed by the gum on the AFM silicon tip. It is of the utmost importance to select an appropriate DCG dose that guarantees a desirable coatability (i.e., adhesion and adsorption) between the binder and mineral aggregates without adversely affecting the resultant mixture performance and functionality.

### 3.6. Conventional Binder Tests (Penetration, Softening Point, and Viscosity)

**Figure 7:**
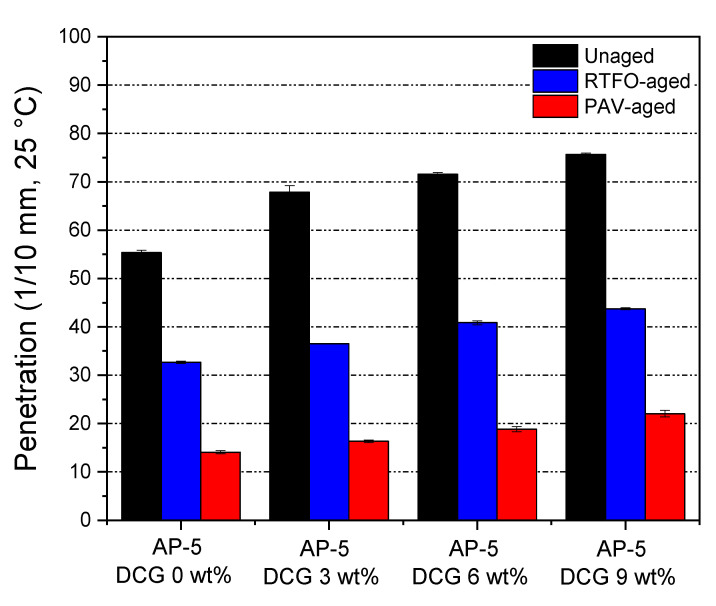
Impact of various dosages of DCG (e.g., 3, 6, 9 wt%) on the penetration of base AP-5 asphalt before and after RTFO and PAV aging.

**Figure 8:**
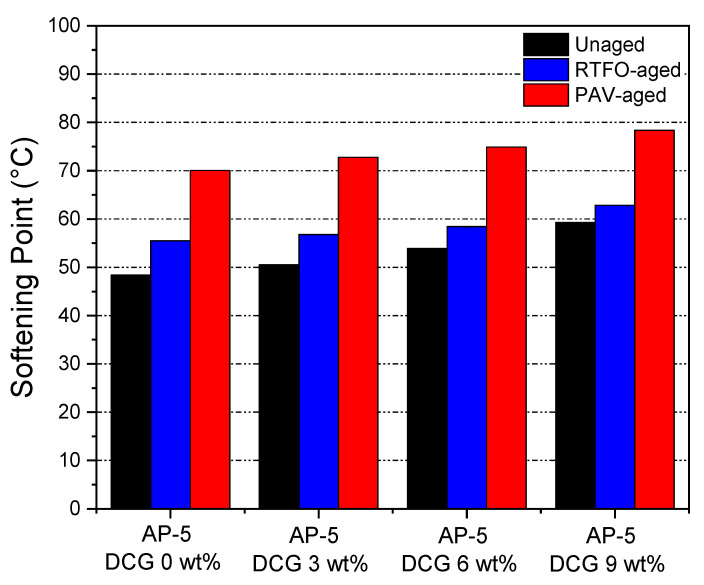
Impact of various dosages (e.g., 3, 6, 9 wt%) on the softening point of base AP-5 asphalt before and after RTFO and PAV aging.

**Figure 9:**
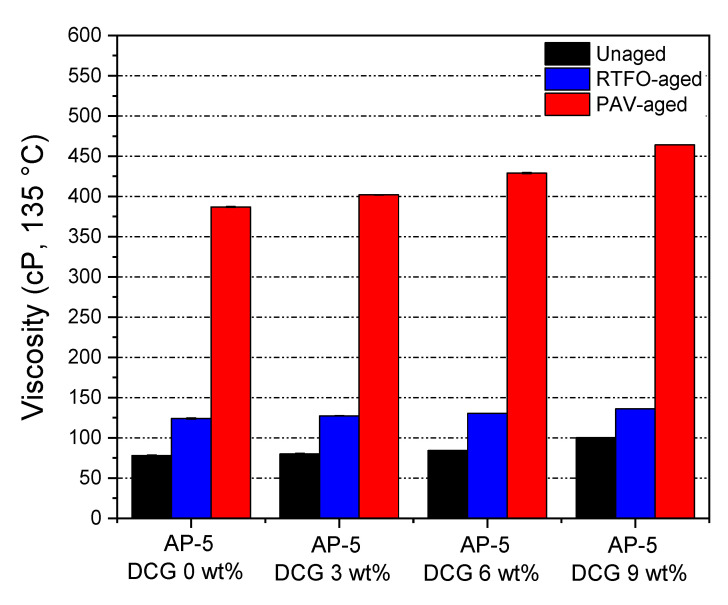
Impact of various dosages of DCG (e.g., 3, 6, 9 wt%) on the viscosity of base AP-5 asphalt before and after RTFO and PAV aging.

Laboratory tests on both unaged and RTFO- and PAV-aged asphalt samples blended with various concentrations of discarded chewing gum (e.g., 3, 6, 9 wt% DCG) were performed to determine the physical and rheological properties of binder. The tests included penetration, softening point, and viscosity. Figure 7, Figure 8 and Figure 9 recapitulate the test results on straight-run (non-modified) and oxidized bituminous samples.

In Figure 7, one can easily notice that the incorporation of waste gum magnified penetration value to certain level, as copolymer diminishes the stiffness of unaged and aged asphaltaneous specimens. This means that the additive along with artificial weathering renders the modified binder softer and less consistent. The higher penetration value may be linked to the gum characteristics, i.e., it tends to swell/solvate; hence, it may have a lower density than that of the base asphalt and therefore, it may be effortlessly penetrated. This is desirable in one sense because it can boost the anti-fatigue cracking performance of mixtures; however, it can detrimentally impact the flexibility-consistency property of the bitumen and make it much tender, thereby affecting its resistance against rutting. Particular attention should be paid to the DCG amount when blended with the binder.

The results of the ring-and-ball technique are listed in Figure 8. The softening point test demonstrates the temperature at which the binder softens. It can be seen that the ring-and-ball softening point temperatures (T_R&B_) increase gradually due to the steady incorporation of the polymeric additive into the bitumen. This phenomenon points out that the binder resistance against heat effects is greatly enhanced and it will restrain its propensity to soften or bleed in hot climate regions. Consequently, the blends will be less vulnerable to temperature swings. The T_R&B_ increased swiftly with the inclusion of 9 wt% of DCG, as shown in Figure 8. Beyond this point, the elastomeric phase of polyvinyl acetate (PVAc) could become continuous, thereby leading to the progressive elevation of T_R&B_. The DCG concentration should be adequate to achieve approximately a T_R&B_ within the 60–80 °C range, which is the maximum temperature usually determined in road pavements. Using a base gum with a fusion temperature larger than 50 °C means that at lower temperatures, it will not fluidify the binder, but instead will aid to stiffen it. Figure 8 indicates that the base AP-5 asphalt which has a low softening point is less vulnerable to rutting in comparison with other binders which possess higher softening points.

A rotational viscosity test (i.e., Brookfield rotational viscometer) was employed to determine the flow characteristics of different unaged and aged polymerized asphalt mixtures to ensure that they can be easily pumped and handled at the hot-mix asphalt facilities and/or during the construction process. The results of the tests are plotted in Figure 9. As can be observed, the incremental DCG addition contributed to the moderate increase in asphalt viscosity, and therefore, the manufacturing and compaction temperatures are not greatly affected. This could be considered as one of the major advantages of DCG/asphalt mixtures. The copolymer modification can deliver greater fluidity and workability within the permissible range of viscosity (i.e., less than 3000 cP at 135 °C). As 9 wt% waste gum is included into the pure asphalt cement, there is a notable rise in the viscosity, as shown in Figure 9. This rise could contribute to the significant dose-concentration introduced as well as to the low melting point of base gum, where it is already in liquid state and may attain a rigid network at 135 °C or higher temperatures. Under the influence of polymer and thermal conditioning, the asphaltene content could also be responsible for the viscosity amplification.

The RTFO- or PAV-aging can stiffen the binder, thereby resulting in a decrease in penetration, and an increase in softening point and viscosity as compared to the thermally unconditioned bituminous specimens. During the accelerated aging process, the asphalt will be subjected to oxidation and loss of volatile compounds. Referring to Figure 7 and Figure 8, it can be noted from the relative growth in penetration and softening point values of aged asphaltaneous samples that the higher thermally stable-DCG is endowed with some anti-aging properties that can assist the pavement in resisting rutting at high service temperatures and resisting fatigue at intermediate and low service temperatures.

### 3.7. Temperature Susceptibility (TS)

Owing to its physical nature as a thermoplastic material, the bitumen’s consistency varies with temperature. Temperature susceptibility (TS) refers to the rate at which the binder’s consistency changes with a fluctuation in temperature, and this is a very essential attribute of asphalt cement.

To specify the TS of non-aged and aged asphaltaneous samples comprising diverse portions of discarded chewing gum (e.g., 3, 6, and 9 wt% DCG), penetration index (PI) and penetration viscosity number (PVN), were adopted.

**Figure 10:**
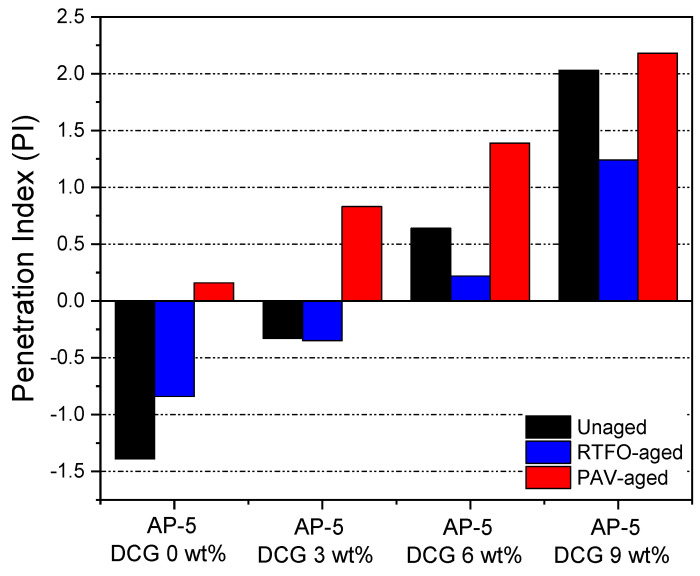
Influence of various fractions of DCG (e.g., 3, 6, 9 wt%) on the penetration index (PI) of base AP-5 asphalt before and after RTFO and PAV aging.

It is pertinent to note from Figure 10 that the PI values range between −1.39 and +2.18. The ideal PI value for better performing roadways should be confined within +1 and −1 range; however, if the value jumps downward to −2, the road pavement will be more prone to the adverse impacts of temperature change and will show greater tendency to induce brittle failure. Bitumina with an index higher than +1 are characterized by a low TS and a slight brittleness [33]. The lowest PI (−0.33) value was achieved by mixing 3 wt% of DCG with the base AP-5 asphalt under unaged conditions; whilst, the highest PI (+2.03) was gained by the unaged original asphalt treated with 9 wt% DCG. In comparison to other bituminous samples, the Newtonian straight asphalt (i.e., AP-5 DCG 0 wt%) will probably display brittleness and would be more vulnerable to transverse cracking in cold climates.

**Figure 11:**
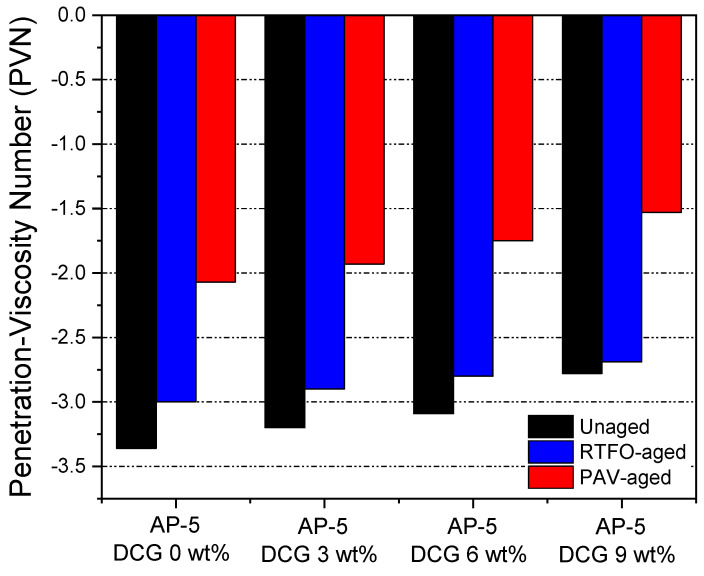
Influence of various fractions of DCG (e.g., 3, 6, 9 wt%) on the penetration viscosity number (PVN) of base AP-5 asphalt before and after RTFO and PAV aging.

The calculated PVN values for the gum-treated asphalts are shown in Figure 11. The penetration-viscosity number values vary between −3.36 and −1.53. Normally, most paving grade bitumina hold a PVN between −2.0 and +0.5. The more positive or larger the PVN, the less temperature-susceptible the asphalt binder will be [33]. The −2.78 PVN, obtained by combining a 9 wt% of DCG with the straight AP-5 asphalt under unaged condition, has a very low TS and should manifest a great tolerance to low temperature milieus without causing transverse pavement cracking. Such binders would necessitate relatively high temperatures to achieve a mixing viscosity. Collectively, it seems reasonable to assume that the use of waste gum will apparently engender a great improvement in the engineering performance of road pavement during the winter season; meanwhile, the bitumen will be capable of combatting cold weather.

### 3.8. Dynamic Shear Rheometer (DSR) Test

To reveal the rutting potential of unmodified and DCG-modified asphalts, DSR test was executed on the polymer/binder mixtures before and after short-term aged condition (i.e., rolling thin film oven test, RTFOT).

**Figure 12:**
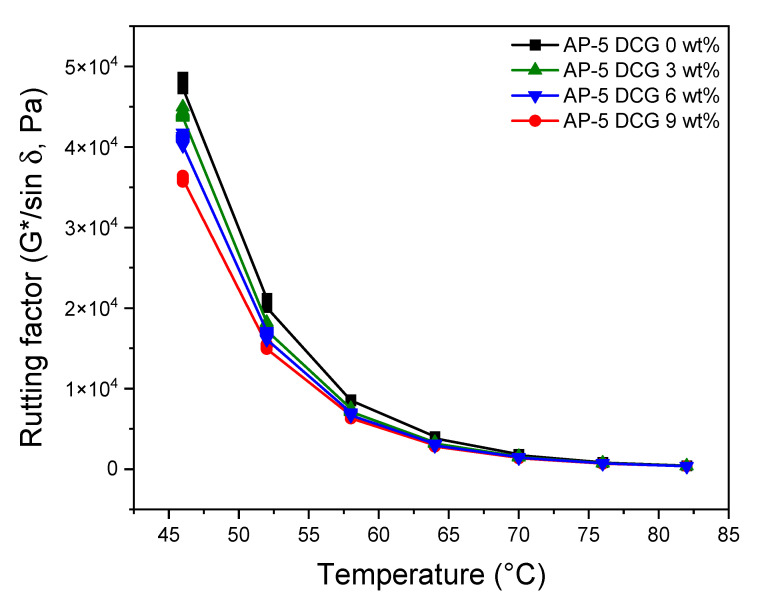
Variation of rutting factor (G*/sin δ) as a function of temperature for unaged base AP-5 asphalt and its modified specimens comprising various doses of DCG (e.g., 3, 6, 9 wt%).

**Figure 13:**
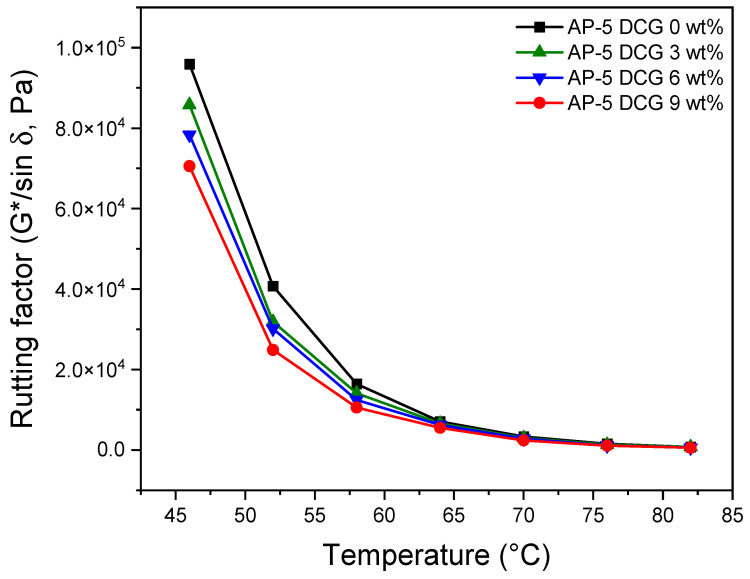
Variation of rutting factor (G*/sin δ) as a function of temperature for RTFO-aged base AP-5 asphalt and its modified specimens comprising various doses of DCG (e.g., 3, 6, 9 wt%).

Figure 12 and Figure 13 summarize the rutting resistance data obtained under unaged and short-term aged conditions. At first glance, it can be noticed from Figure 12 that the unaged original AP-5 asphalt (i.e., AP-5 DCG 0 wt%) is characterized by a higher rutting potential in comparison to unaged DCG-modified asphalt specimens (i.e., AP-5 DCG 3, 6, and 9 wt%). Additionally, the stiffness parameter showed a moderate drop with the increase in DCG concentration and testing temperature. Below 58 °C, all the asphalt samples met the minimum requirements (i.e., G*/sin δ ≥ 1.0 kPa) of the Superpave Construction Guidelines [34]. The neat asphalt molded with 3, 6, 9 wt% DCG fulfilled the requirements at 70 °C. This temperature actually corresponds to the maximum temperature where the unmodified and modified asphalts can reach good viscoelastic performance once introduced into the pavement.

Figure 13 shows the effects of DCG on the rutting index of RTFO-aged asphalt samples as a function of temperature. Again, the rutting performance of blends decreased moderately as the level of gum dosage and testing temperature increase. The asphalt binders treated with 3, 6, and 9 wt% are only able to resist rutting up to ca. 70 °C (i.e., G*/sin δ ≥ 2.2 kPa) [34]. A slight decrement in G*/sin δ reveals moderate resistance against rutting in the high-temperature regime. The most striking reduction appears with the addition of 9 wt% DCG. Using more than 9 wt% DCG may not be economically viable because of the limited advantages on lowering rutting.

The overall data suggest that using discarded chewing gum as a bitumen modifier or additive can moderately alter rutting in newly constructed pavements. The gradual degradation of resistance to permanent deformation by DCG could mainly be attributed to the molecular weight and the content of polyvinyl acetate (PVAc) within the base gum.

Here, it should not be forgotten that discarded chewing gum is made up of a mixture of components varying between resins, waxes, and elastomers. As mentioned before, resin is the key chewable fragment, whereas wax aids to soften the gum, and elastomers add flexibility. Other components may be equally present in the form of residues, particularly food-grade softeners, sweeteners, preservatives, flavorings, fillers, and even salivary enzymes. This complex material, when added to the binder, will definitely affect its original characteristics and behaviors. For instance, when the gum–bitumen blend is subjected to any chemical, thermal, or physico-rheological treatment, each single ingredient or additive embedded in the gum matrix will react or interact in its own way depending on its innate and acquired properties, reaction medium, internal and external factors. Therefore, the performances achieved by the DCG-modified asphalt specimens might not always be in harmony between testing methods such as the softening point test and rutting factor test, where the obtained data are surprisingly contradictory. This is an important point which deserves further attention in order to identify the real causes behind this discrepancy.

**Figure 14:**
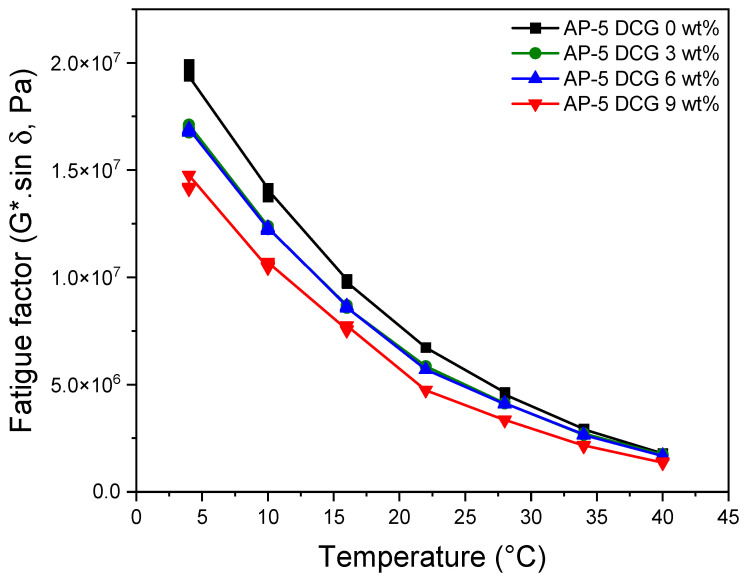
Variation of fatigue factor (G*.sin δ) as function of temperature for PAV-aged base AP-5 asphalt and its modified specimens comprising various doses of DCG (e.g., 3, 6, 9 wt%).

To unveil the fatigue cracking potential of untreated and DCG-treated asphalts, the DSR test was performed on the polymer–binder blends after long-term aged condition (i.e., pressure-aging vessel, PAV). As portrayed in Figure 14, G*.sin δ values are steadily decreased with the increase in the DCG fraction and testing temperature, with respect to the base bitumen. Owing to the in-situ crosslinking reactions (i.e., molecular entanglements) happened between the binder and polymer, the lower G*.sin δ value reveals less shearing energy loss and better capacity of the fatigue resistance. All asphalt samples treated with 3, 6, 9 wt% DCG passed the qualification of Superpave specifications (i.e., G*.sin δ ≤ 5000 kPa) [35] at 28, 25, 25, and 22 °C, respectively. It can be inferred that DCG incorporation into the neat asphalt can potentially enhance its resistance potential to fatigue cracking at intermediate temperatures. 

### 3.9. Performance Grade (PG) Test

The Dynamic Shear Rheometer (DSR) data and Superpave Performance Classification system [51] are utilized to diagnose the performance grade (PG) of the base AP-5 asphalt along with its samples modified with various percentages of discarded chewing gum (e.g., 3, 6, 9 wt% DCG).

**Figure 15:**
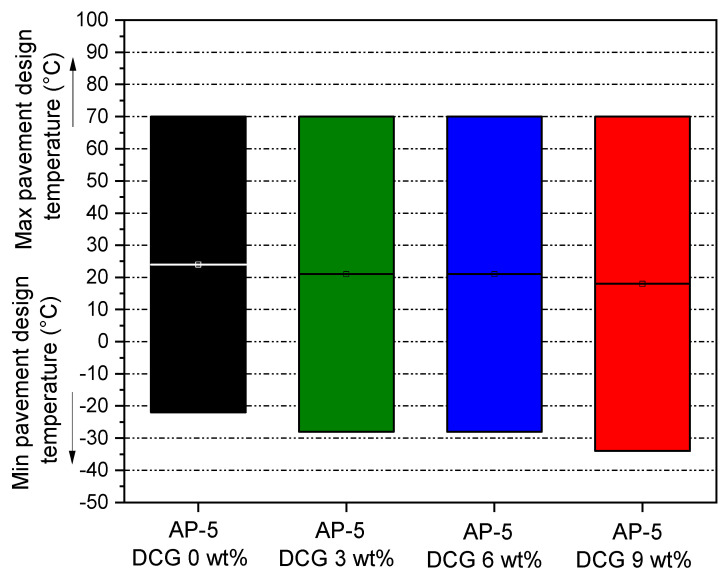
Impact of various amounts of DCG (e.g., 3, 6, 9 wt%) on the performance grade (PG) of base AP-5 asphalt.

The impact of DCG on PG of asphalt binder is shown in Figure 15. The PG 70–22 means that the straight AP-5 asphalt offers a satisfactory performance at common traffic loads in the temperature range from −22 to 70 °C. Generally, the PG system describes the climate-related optimal operating conditions of a given binder through specifications for low and high temperatures characteristics of bitumen that correlate to road pavement performance.

With respect to the neat binder, the modification influence of DCG is seemingly negligible at elevated temperatures, which demonstrates a non-improvement of rutting resistance through grade bumping upward by 0 grade (i.e., one grade equivalent to 6 °C) at higher temperatures. On the other hand, the waste gum is found to cause a remarkable rise in PG at colder temperatures through grade bumping downward by +1 grade for AP-5 DCG 3 and 6 wt% and by +2 grades for AP-5 DCG 9 wt%, thereby indicating that there is a notable stiffening effect on the treated bitumen at lower temperatures by reducing critical low-temperature for thermal cracking compared to the virgin bitumen.

Taken as a whole, the outcomes of the binder tests assert that the discarded chewing gum examined in this research project could not significantly boost the upper performance of bitumen which will unfavorably assist the roadways to resist rutting in hot climate regions; however, it could greatly enhance the lower performance grade by aiding the pavement to withstand cracking potential in cold climate regions.

### 3.10. Thermogravimetric Analysis (TGA/DTGA)

**Figure 16:**
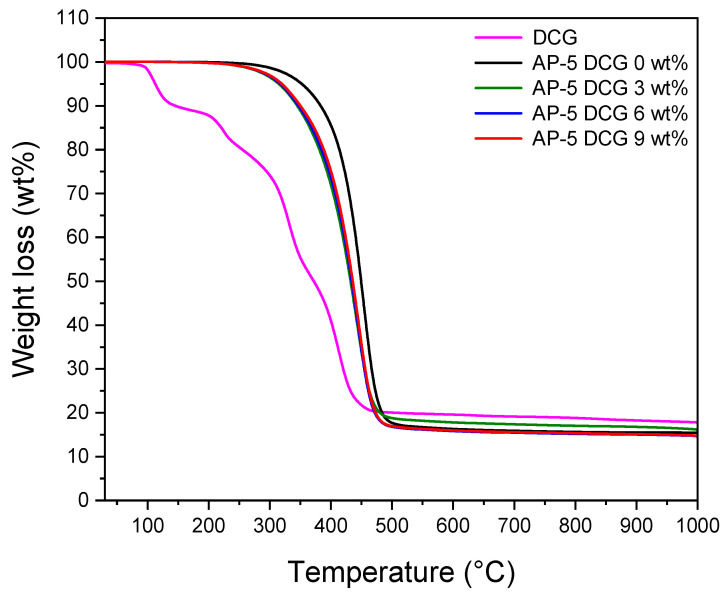
TGA thermograms of DCG, base AP-5 asphalt, and its specimens containing various fractions of DCG (e.g., 3, 6, 9 wt%).

**Figure 17:**
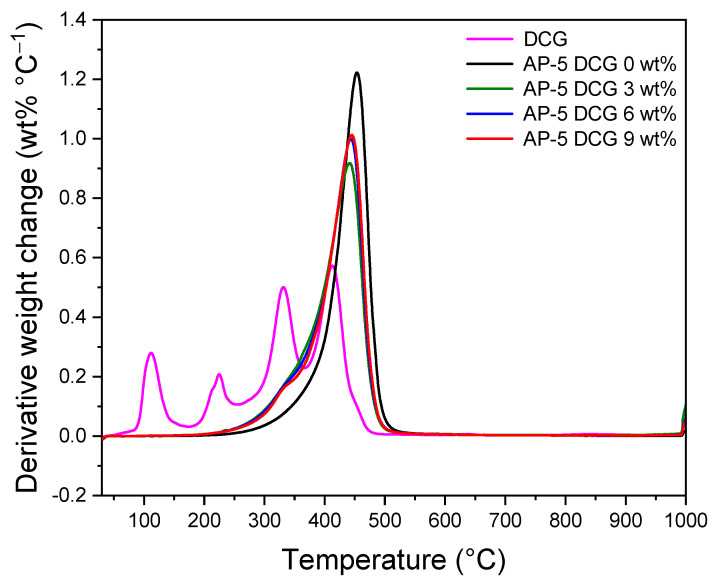
DTGA thermograms of DCG, base AP-5 asphalt, and its specimens containing various fractions of DCG (e.g., 3, 6, 9 wt%).

Thermogravimetric analysis (TGA) was utilized to gain in-depth knowledge about the thermal and/or oxidative stabilities and compositional properties of the base asphalt AP-5 after its modification with different portions of discarded chewing gum (e.g., 3, 6, 9 wt% DCG). The primary and derivative thermograms for the DCG, virgin bitumen, and DCG-modified asphalt samples under nitrogen purge and temperatures of 30–1000 °C, are given in Figure 16 and Figure 17, respectively.

The TGA of DCG shows that the mass loss occurs in four distinct stages, which confirms that the gummy material under investigation has multiple components. The thermal stability of the gum waste is an interesting feature that could make the material fit for road pavement applications where the binder is thermally processed using hot-mix asphalt plant operations. Referring to Table 3, it is apparent that the incremental addition of gum into the virgin bitumen barely altered its thermal stability (i.e., T_onset_ of thermal degradation); however, this could be restored progressively by further incorporation of waste chewing gum and could greatly boost the heat resistance of binder by adopting larger modifier concentration (greater than 9 wt% DCG).

Figure 16 displays also the weight loss of virgin base asphalt AP-5 (i.e., AP-5 DCG 0 wt%) as a function of temperatures. This figure shows that the onset temperature of the main loss effect is T_onset_, 409.25 °C. Three main temperature ranges are observed as three phases of weight loss: 30.11–409.25, 409.25–476.79, and 476.79–999.98 °C. The first weight loss is mainly related to the decomposition and volatilization of saturate (5.08 wt%) and aromatic (15.30 wt%) compounds. The second weight loss is primarily attributed to the decomposition of resins (57.16 wt%) along with few asphaltene molecules. The chemical reactions that may have occurred are decomposition, oxidation, and reduction; they are more complex and severe in this mass loss phase. The aromatic compounds are still disintegrated within this range of temperature [52]. The third weight loss is related to the complete degradation of asphaltenes (22.48 wt%). Finally, approximately 15.35 wt% of carbon depending on the heating rates was remained as a charred residue after heating at 999.98 °C.

The DTGA thermogram of plain bitumen, given in Figure 17, shows, interestingly, a single step decomposition pattern with a maximum temperature at 453.65 °C, which is an indication that the pure asphaltaneous product understudy is rich in resin materials (57.16 wt%). This finding is supported by TLC-FID analysis.

### 3.11. Differential Scanning Calorimetry (DSC)

**Figure 18:**
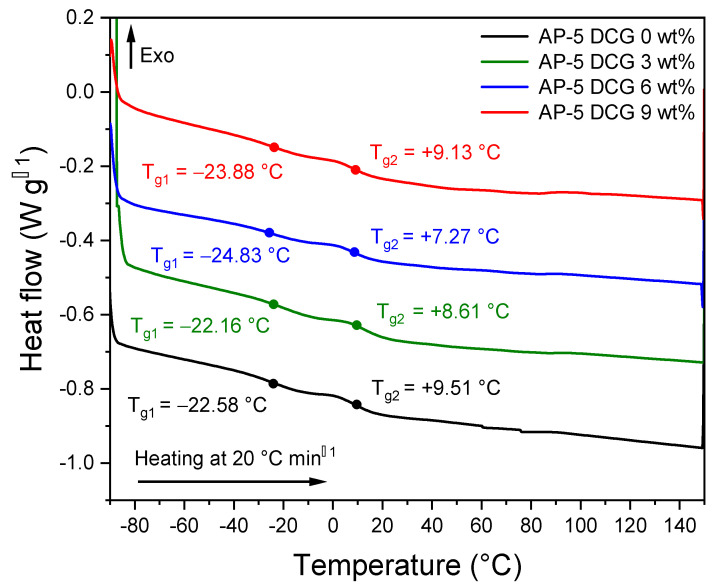
DSC thermograms of base AP-5 asphalt and its specimens loaded with various fractions of DCG (e.g., 3, 6, and 9 wt%).

**Figure 19:**
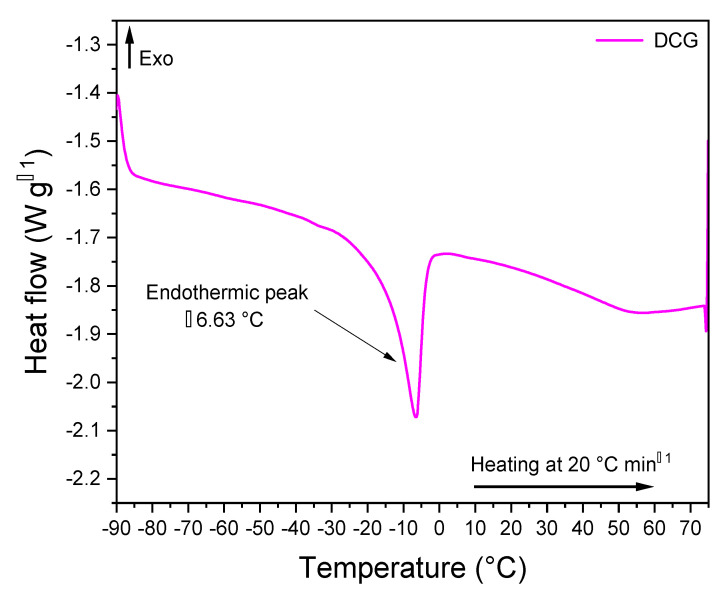
DSC thermogram of discarded chewing gum (DCG).

To study the effect exerted by the discarded chewing gum (DCG) on the thermal properties and behaviors of base asphalt AP-5, the differential scanning calorimetry (DSC) was carried out. Figure 18 shows a compilation of thermograms distributed between untreated and DCG-treated asphalt samples, while Figure 19 portrays the DSC thermogram of DCG.

Referring to Figure 18, all asphalt specimens display two narrow glass transition temperatures. Similar to most bitumina, AP-5 asphalt is seemingly heterogenous rather than a homogenous system. It can be plainly seen that the straight binder (i.e., AP-5 DCG 0 wt%) exhibits two visible thermal transitions or typically two glass transition temperatures (T_g1_ and T_g2_) located approximately at −22.58 and +9.51 °C, respectively. T_g_ rises naturally with the stiffness, aromaticity, polarity, and molecular weight of the repeat molecular structure within the amorphous phase [53].

Generally, typical bitumina exhibit four distinct glass transition temperatures that originate from various amorphous phases within their matrices: the first one (T_g Sa_) is allocated to saturates (i.e., *n*- and *iso*-alkanes) and can occur within the −88~−60 °C range. T_g Sa_ should emerge from a phase rich in flexible paraffinic segments. The second one (T_g Ma_) at −20 °C, which is the most pronounced transition, stems principally from the maltene phase (i.e., saturates, aromatics, and resins). The third glass transition temperature (T_g Ma-As_) around −10 °C is assigned to the maltene–asphaltene interphase region of mixed composition, likely rich in resin. The fourth one (T_g As_) arises mostly from asphaltenes (i.e., alkylated condensed aromatic rings) and can appear at approximately +70 °C [54,55,56,57,58].

Eventually, no exothermic peaks above the third glass transition temperature (T_g Ma-As_) were detected, which ordinally originates from the crystallization of wax (i.e., small paraffin molecules) occurring during the heating process [59]. Similarly, no endothermic event was detected, during the scan test, which is usually due to the melting of crystallizing fractions. This could be related to the insignificant amount of saturates, a mixture of *n*-alkanes, found in AP-5 asphalt.

The thermal behavior of asphalt binders is extremely complicated, and depends mainly on their origins, the processes by which they are manufactured, their thermal history, and so on [60]. This fact could plausibly explain the several inconsistencies found between the T_g_ values of this study and those reported in the literature. However, much work still needs to be undertaken to carefully understand the obtained findings.

Comparisons of the DSC scan data indicate that the addition of 6 or 9 wt% waste gum into the neat binder shifted its glass transition temperatures downward, possibly enhancing its low-temperature performance [61].

The DSC curve of discarded chewing gum (DCG) is displayed in Figure 19. This curve shows a single sharp melting endothermic transition between −28 and +2.50 °C with a centered peak at T_m_ = −6.63 °C and a fusion enthalpy (∆H_DCG_) of 18.04 J/g. The melting temperature (T_m_) corresponds to the fusion temperature of amorphous polyvinyl acetate (PVAc). The gum base is a mixture of polymers; the most commonly used polymer in chewing gum products is polyvinyl acetate (PVAc), which can be used as a resin, plasticizer, or elastomer solvent [62]. It should be mentioned that PVAc generally exhibits two glass transition temperatures: the first one (T_g1 PAVc_) could be detected around +5.65 °C, whereas the second one (T_g2 PAVc_) could be identified at +17.50 °C. This temperature range plays a crucial role during chewing gum processing in order to attain appropriate hardness for coating. Additionally, the PVAc thermogram should reveal one prominent fusion band around +50.90 °C, associated with a change in heat capacity (Cp). This endothermic band is also referred to as the “Drop Softening Point” by the chewing gum industry, where the gum’s viscosity normally falls in the range 60–80 °C [62].

Unexpectedly, no glass transition temperature was found in the DCG case, while the melting temperature was substantially shifted to lower values (ca. −6.63 °C). It has been reported that when any ingredient is added to PVAc (e.g., sugar, starch mix, elastomers such as polyisobutylene (PIB), etc.); the peak position and shape could have great tendency to negatively displace or disappear gradually, respectively [62]. Additionally, the addition of plasticizers such as glycerin to the gum base could significantly alter its glass transition temperature [63]. Hence, the peak with a maximum heat absorption at −6.63 °C is most likely related to the PVAc entrapped in the gum base along with other additives and residual saliva (N.B. salivary enzymes could certainly affect the thermal properties of chewing gum).

No crystallization event was observed because PVAc and elastomers in the mixture are non-crystalline or amorphous polymers with more molecular mobility, where crystallization cannot be perceived. Increasing the mobility of the molecules by means of the addition of low-molecular weight compounds or plasticizers should assist the overall bitumen performance, by improving its flexibility and cohesivity. Likewise, in the case of polymer–asphalt mixtures, the low glass transition temperature can boost low-temperature properties [64], thereby improving workability in cold weather construction.

## 4. Conclusions

In reviewing the data presented in this research project, it is clear that the discarded chewing gum (DCG) is ideally suited for use as a superior asphalt additive for road construction and paving. It is easier to blend, has a great miscibility with the binder, and offers improved fatigue cracking resistance as witnessed by DSR test. Indeed, it can enlarge the functional temperature range of asphalt cement, thereby allowing the production of several performance-graded bitumina. On the basis of Iatroscan TLC-FID data, it was found that the aromatics and resins behaved differently in response to the steady incorporation of waste gum into the binder; however, there was no notable change in the content of saturate and asphaltene fractions. Furthermore, the interesting talc mineral found with the help of FT-IR and XRD techniques throughout the blend matrix can be exploited as a filler to greatly improve the mechanical strength properties of asphalt paving mixtures. XRD analysis demonstrated that the discarded chewing gum marginally affected the amorphous phase of AP-5 base asphalt, but its microcrystalline phase did not. FT-IR scan highlighted that the modification of straight asphalt with DCG was physical and not chemical. Visual inspections made using SEM and AFM techniques pointed out that incremental addition of the chemical intact-DCG into the plain bitumen provoked a considerable growth in the roughness rate, while expanding the size of bee-shaped microstructures, contracting the *peri*-phase domains, and abolishing the *para*-phase domains. Traditional physical empirical tests indicated that the waste gum could also bring excellent processability of the asphalt mix by almost retaining its viscosity, whilst increasing penetration and softening point values. TGA/DTGA/DSC studies revealed that the additive altered the thermal behaviors and properties of original binder slightly. In addition to its higher thermal stability, DCG could impart the bituminous blends with myriad benefits, including improving aging process resistance, reducing thermal susceptibility, avoiding binder from extensive oxidation, and providing sufficient elasticity, thereby preventing cracking in cold weather. As a whole, the use of DCG as a bitumen modifier is economically viable and can appreciably contribute in tackling plastic pollution, while boosting hot-asphalt mix performance and delivering longer-lasting roads. Giving special attention to rutting distress factor and adopting a special preprocessing to the gum waste before use would effectively ensure achieving an outstanding performance. Finally, it is highly recommended to conduct some additional laboratory tests, including but not limited to the compatibility test, bending beam rheometer test, storage stability test, toughness and tenacity test, wheel-tracking test, direct tension test, etc., to enable effective assessment of the promising additive before applying it in real-time and large-scale pavement projects.

## Figures and Tables

**Table 1:** Physicochemical properties of base AP-5 asphalt.

Elemental Analysis	Mean ± SD
C (carbon)	83.40 ± 0.04 wt%
H (hydrogen)	10.03 ± 0.02 wt%
N (nitrogen)	0.44 ± 0.01 wt%
S (sulfur)	5.46 ± 0.02 wt%
O (oxygen)	0.49 ± 0.01 wt%
**SARA Generic Fractions**	
Saturates	5.08 ± 0.51 wt%
Aromatics	15.30 ± 1.28 wt%
Resins	57.16 ± 1.04 wt%
Asphaltenes	22.48 ± 1.18 wt%
**Physical Properties**	
Penetration at 25 °C	55.33 ± 0.51 dmm
Softening point	48.37 ± 0.02 °C
Rotational viscosity at 135 °C	78 ± 0.89 cP
Density at 25 °C	1.00 ± 00 g/cm^3^

**Table 2:** Chemical characteristics of discarded chewing gum (DCG).

Elemental Analysis	Mean ± SD
C (carbon)	60.27 ± 0.62 wt%
H (hydrogen)	9.63 ± 0.08 wt%
N (nitrogen)	0.10 ± 0.00 wt%
S (sulfur)	0 ± 0.00 wt%
O (oxygen)	16.51 ± 0.13 wt%
**SAR Ingredients**	
Saturates	17.98 ± 1.14 wt%
Aromatics	22.93 ± 1.07 wt%
Resins	59.08 ± 1.57 wt%

**Table 3:** TGA and DTGA thermogram data of DCG, base AP-5 asphalt, and DCG-AP-5 mixtures comprising 3, 6, and 9 wt% DCG loading at a heating rate of 20 °C min^−1^.

Sample	TGA/DTGA (°C)						−ΔW (wt%)
	Stage 1	Stage 2	Stage 3	T_onset_	T_offset_	T_max_	
DCG	30.19~367.54	367.54~438.07	438.07~999.87	311.22	438.07	412.71	17.65
AP-5 DCG 0 wt%	30.11~409.25	409.25~476.79	476.79~999.98	409.25	476.79	453.65	15.35
AP-5 DCG 3 wt%	30.13~378.96	378.96~467.22	467.22~999.93	378.96	467.22	441.93	15.76
AP-5 DCG 6 wt%	30.11~385.12	385.12~468.22	468.22~999.95	385.12	468.22	443.82	14.45
AP-5 DCG 9 wt%	30.12~387.38	387.38~469.26	469.26~999.94	387.38	469.26	445.40	14.59

T_onset_, onset of thermal degradation (°C); T_offset_, offset of temperature of final loss (°C); T_max_, maximum decomposition temperature (°C); ΔW, carbonaceous residue at 1000 °C (wt%).

## Data Availability

The data presented in this study are available on request from the corresponding author.
